# The Goldilocks Model for TCR—Too Much Attraction Might Not Be Best for Vaccine Design

**DOI:** 10.1371/journal.pbio.1000482

**Published:** 2010-09-14

**Authors:** Jill E. Slansky, Kimberly R. Jordan

**Affiliations:** University of Colorado Denver School of Medicine, Denver, Colorado, United States of America

## Abstract

Recent research on T cell-antigen interactions suggests that tighter binding is not always better at eliciting an effective immune response.

One of the most exciting avenues of translational research today is determining how to program αβ T cells of the immune system to treat diseases such as cancer. The main function of T cells is the surveillance of unhealthy cells throughout the body. Because of their specificity, function, and lasting memory, T cells can contribute to the prevention and treatment of many diseases. Two approaches that are being explored to improve T cell responses using different vaccine methods are adoptive cell therapies, in which T cells are expanded and possibly manipulated ex vivo and then reintroduced into the patients [1], and stimulation of the endogenous T cell repertoire [2]. Both hold promise for antigen-specific treatments of diseases.

## The TCR–Peptide–MHC Interaction

The specificity of the interaction of T cells with their targets is provided by the T cell receptor (TCR). A limitless number of different receptors are generated by somatic recombination during T cell development [3]. Each T cell develops a unique receptor sequence, which can interact specifically with different targets. The consequence of this interaction depends on many events that take place during T cell development. In addition to the TCRs, the T cells express co-receptors that also interact with the target cells. Traditionally, T cells that express the cluster of differentiation (CD) 8 co-receptors are cytotoxic, and T cells that express the CD4 co-receptors help to orchestrate the immune response by either activating or dampening the response of other immune cells.

The molecular target of the TCR is a peptide antigen bound to an antigen-presenting molecule found on the surface of most cells in the body that is known as a major histocompatibility complex (MHC) molecule. The peptide sits in the MHC molecule like a hotdog in a bun so that the surface of the peptide-MHC complex is available for interactions with TCRs [4],[5]. Self proteins, mutated or oncogenic proteins, or pathogen-derived proteins are processed and cleaved into short peptides. The peptides derived from proteins normally found inside the cell are conventionally presented to CD8+ T cells by MHC class I molecules, and those engulfed from the extracellular milieu are conventionally presented to CD4+ T cells by MHC class II molecules.

The TCR–peptide–MHC interaction leads to a spectrum of T cell responses. Immature T cells that interact with MHC molecules are selected for further maturation during positive and negative selection in the thymus [6],[7]. Those that are strongly reactive towards self antigens are eliminated during negative selection. T cells that are not negatively selected, but interact with high affinity ligands may develop into regulatory T cells, which suppress immune responses [8]. The remaining pool of T cells interact with peptide-MHC molecules during an immune response, triggering signals that are propagated though the TCR into the cell and leading to cell division. The fate of T cells depends on the strength of the interactions and the surrounding environment. For example, a number of studies using CD4+ TCR transgenic T cells show that when the sensitivity of the TCR for an antigen is changed, the cytokines produced by the T cells also change [9],[10]. Thus, how well T cells interact with target cells and the conditions under which they interact are both important.

## The TCR–Peptide–MHC Interaction in Basic Immunology

Different, non-mutually exclusive models developed in the last 15 years help describe the optimal affinity of the TCR–peptide–MHC interaction. The kinetic-proofreading model proposes that T cells cannot be fully activated unless the TCR–peptide–MHC interaction remains engaged long enough for the necessary signaling events to take place [11]. Another model is the serial-triggering model, in which one peptide–MHC complex binds multiple TCRs to amplify and sustain signaling by the T cell [12]. A third model predicts that there is an upper and lower limit to the half-life of binding, or the dwell-time, of the TCR–peptide–MHC interaction, which narrows the range of affinities that lead to productive interactions [13]. In addition, the CD8 and CD4 co-receptors may augment [14] or inhibit [15] the apparent affinity of the TCR–peptide–MHC interaction. Recent results published by Huppa et al. have examined the TCR–peptide–MHC interaction on cells rather than as proteins in solution [16]. They show that the CD4 co-receptor engagement does not contribute to the physical association, but is required for optimal signaling into the T cell. The affinity of many TCR-peptide-MHC interactions has been determined without co-receptor binding, and most natural TCRs fall in the 1–200 µM range [17],. These affinities are weaker than many other protein–protein interactions: antibody–antigen interactions are usually in the nanomolar range, whereas avidin–biotin affinity is in the femtomolar range. What, then, is the “ideal” affinity for TCRs, and can it be manipulated to optimize therapeutic immune responses?

## The TCR–Peptide–MHC Interaction in the Potency of Vaccines

**Figure 1:**
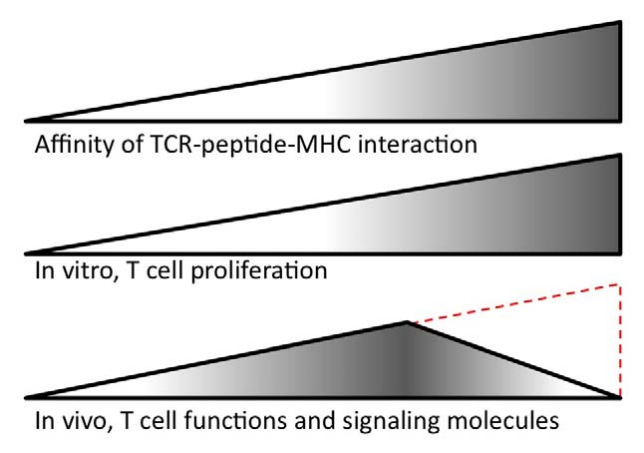
Summary of Corse's findings and the controversy. Corse and colleagues studied peptides that bind with increasing affinity in the TCR-peptide-MHC interaction. In vitro, T cell response increases as the affinity increases. In vivo, T cell function and expression of signaling-related molecules peaks at an intermediate affinity. The same CD4+ T cells from a transgenic mouse were used in both the in vitro and in vivo experiments. These results contrast with those from related studies using different types of T cells and experimental conditions. Specifically, Zehn and colleagues examined how stimulation of CD8+ T cells from a transgenic mouse responded to cognate peptides with increasing affinity [20] and found a direct correlation between affinity and the number of T cells, as indicated in red. (Signaling-related molecules were not examined in this study.)

Significant interest lies in understanding the optimal affinity range in the TCR–peptide–MHC interaction for vaccine design. The strength of this interaction, in addition to the other factors that contribute to this interaction (Table 1), determines the outcome of the T cell response. Research by Emily Corse and colleagues, published in this issue of *PLoS Biology*, used a simple system to determine the affinity range that activates T cells by vaccination (Figure 1). They used a panel of four related peptides that bind to a mouse MHC class II molecule [19] and CD4+ T cells from a TCR transgenic mouse [9]. Using standard methods to determine the binding affinity of TCR–peptide–MHC interactions, they show that the affinity of these peptides varies across the group. Although the two highest affinity peptides bind similarly when comparing the monomeric interaction by surface plasmon resonance, they differ significantly when comparing the intensity of staining with multivalent ligands (i.e., staining with MHC tetramers). Although determining the monomeric interaction provides the relative binding properties, generally multimeric binding is more physiologically relevant because there are numerous interactions between two cells. The other two peptides are weaker in both assays. The hierarchy of in vitro stimulation of cognate T cells with these peptides correlates with the affinity values.

**Table 1:** Some factors that influence the strength of T cell responses.

**T cell-related factors**	Interactions between T cells and antigen presenting cells
	• Affinity of the T cell receptor-peptide-MHC interaction
	• Co-receptor binding
	• Co-stimulatory and checkpoint molecule interactions
	• Adhesion molecule interactions
	• T cell receptor expression
	Repertoire and precursor frequency of reactive T cells as a result of positive and negative selection
	T cell receptor down-regulation, internalization, or degradation
	Intrinsic T cell signaling/kinase and phosphatase activity
	Activation-induced cell death of the T cell
	Primary vs. memory response
**Antigen-related factors**	Structure/landscape/available contact points for the T cell receptor
	Presentation of peptide by MHC molecules/peptide-MHC affinity
	Processing of peptides/immunodominance
	Binding register of the peptide within the MHC molecule
	Dose/concentration/density of peptide
**Extrinsic factors**	Adjuvant used during T cell priming
	Regulatory T cells and other peripheral tolerance mechanisms
	Cytokine milieu
	Nutrient and metabolite availability

Surprisingly, this correlation is no longer maintained when the peptides are evaluated in a vaccination setting in vivo. The responses to the highest affinity peptide are blunted relative to the intermediate-affinity peptides. The T cells activated by the high-affinity peptide proliferate less and make less cytokine (IFNγ and IL-2), and fewer of the responding T cells express activation markers (pAkt and pStat-3). Finally, the T cells activated by the highest affinity peptide express less of the PD-1 molecule, a marker of T cell exhaustion.

Occam's razor presumes that the stronger the TCR–peptide–MHC interaction, the stronger the T cell response. In fact, unlike the Corse paper, a previous study by Zehn et al. concluded precisely that [20]. In a similarly clean experimental system, Zehn et al. tested a panel of six related peptides that bind to the mouse MHC class I molecule and CD8+ T cells from a TCR transgenic mouse. In these experiments, the T cells were transferred into a syngenic mouse, and the mice were challenged with *Listeria monocytogenes* expressing the different peptides. Unlike the Corse study, the proliferation of the T cells correlated directly with the affinity of the TCR–peptide–MHC interaction in vitro and in vivo.

What might cause this discrepancy? These two papers use different model systems; Corse et al. used CD4+ T cells and the TCR–peptide–MHC interaction had an affinity range from 42.4 to 165 µM. Zehn et al. used CD8+ T cells and the affinity of the TCR–peptide–MHC interaction was greater than 5.9 µM [21]. The panel of peptides analyzed by Corse et al. had affinities higher and lower than the cognate peptide in that study. The cognate peptide in Zehn's study was at the top of the affinity spectrum, and since they did not analyze peptides of higher affinity, it is possible that the cognate peptide falls in the intermediate range. In addition, the authors measure different outcomes. Because the route and method of vaccination differed, other aspects of the milieu may contribute to the differences, TCR down-regulation may be alternatively regulated, the kinetics of other binding interactions may be involved, or the induction of cell death may differ. Finally, the peptides chosen for each TCR may not cover the entire range of affinities required to produce similar outcomes. Thus, it may be possible that a peptide of relative intermediate affinity may best stimulate T cells using the particular conditions in the Corse study, but until this topic is better investigated it is unclear if all peptides will follow the same model.

Our own lab also analyzed a panel of peptides with a range of affinities to determine which peptides generate the most effective antitumor immunity. As in the studies above, we found that the in vitro functions of antigen-specific CD8+ T cells correlate with the TCR–peptide–MHC binding affinity [22]. However, similar to the Corse study, we observed that the highest affinity peptides are not as effective at eliciting antitumor responses as the intermediate affinity peptides. However, in these experiments we examined the natural T cell response to the peptides, not the response of monoclonal transferred cells. The repertoire of T cells that respond to each of the peptides is different, which makes the corresponding affinity difficult to evaluate. In addition, when we vaccinated mice with the high-affinity peptides, we obtained different results in the presence and absence of the tumor, suggesting that the tumor environment is also influencing the response [23].

## Increasing the Affinity of the Interaction by Mutating the TCR

A number of studies have proceeded on the assumption that increasing the affinity of the TCR–peptide–MHC interaction improves T cell immunity. An alternative approach to changing the peptide, as mentioned above, is to genetically increase the affinity of the TCR for tumor antigens rather than changing the antigen. High-affinity tumor–antigen-specific TCRs have been generated previously by screening phage display libraries, and it was found that the changes in the TCR were focused on regions that interact with both the MHC molecule and the antigenic peptide [24]. These results show that as the affinity of the TCRs increases into the pM range, the TCRs become more specific for the MHC molecule, and less specific for the peptide [25]. The functional consequence of losing peptide specificity is that many different peripheral antigens may activate these T cells, which could lead to indiscriminant killing of innocent or healthy cells. Another group used a similar in vitro evolution method to increase the affinity of a TCR for its cognate antigen and focused the changes in the region of the receptor that interacts with the peptide [26]. This TCR paradoxically had reduced antigen specificity in the presence of the CD8 co-receptor, highlighting the potential influence of co-receptor molecules in the strength of the TCR–peptide–MHC interaction [27].

How then do cells find the right balance (the “Goldilocks” level) between high- and low-affinity interactions? It is likely that the many factors that influence the T cell response in vivo after interaction with a high-affinity ligand are involved. These factors may provide “peripheral negative selection” to potentially protect the host from promiscuous T cells that may otherwise be highly auto-reactive. Toward understanding this possibility and resolving the conflicting findings of studies in different systems, it would be interesting to determine if the blunted high-affinity interactions identified by Corse et al. focused more on the MHC molecule than the peptide and to determine what other changes are taking place to the T cell and surrounding environment. In conclusion, T cells may only naturally function in a narrow range of affinities under most conditions to ensure optimal responses against foreign pathogens and minimal responses against auto-antigens, and there is much yet to be learned about the complex factors that influence TCR–peptide–MHC interactions and their downstream consequences.

## Abbreviations

CD: cluster of differentiation
MHC: major histocompatibility complex
TCR: T cell receptor
